# Circulating Leukocyte Subsets Before and After a Breast Cancer Diagnosis and Therapy

**DOI:** 10.1001/jamanetworkopen.2023.56113

**Published:** 2024-02-15

**Authors:** Jacob K. Kresovich, Katie M. O’Brien, Zongli Xu, Clarice R. Weinberg, Dale P. Sandler, Jack A. Taylor

**Affiliations:** 1 Department of Cancer Epidemiology, H. Lee Moffitt Cancer Center & Research Institute, Tampa, Florida; 2 Department of Breast Oncology, H. Lee Moffitt Cancer Center & Research Institute, Tampa, Florida; 3 Epidemiology Branch, National Institute of Environmental Health Sciences, National Institutes of Health (NIH), Research Triangle Park, North Carolina; 4 Biostatistics and Computational Biology Branch, National Institute of Environmental Health Sciences, NIH, Research Triangle Park, North Carolina; 5 Epigenetic and Stem Cell Biology Laboratory, National Institute of Environmental Health Sciences, NIH, Research Triangle Park, North Carolina

## Abstract

**Importance:**

Changes in leukocyte composition often precede chronic disease onset. Patients with a history of breast cancer (hereinafter referred to as breast cancer survivors) are at increased risk for subsequent chronic diseases, but the long-term changes in peripheral leukocyte composition following a breast cancer diagnosis and treatment remain unknown.

**Objective:**

To examine longitudinal changes in peripheral leukocyte composition in women who did and did not develop breast cancer and identify whether differences in breast cancer survivors were associated with specific treatments.

**Design, Setting, and Participants:**

In this prospective cohort study, paired blood samples were collected from 2315 women enrolled in The Sister Study, a US-nationwide prospective cohort study of 50 884 women, at baseline (July 2003 to March 2009) and follow-up (October 2013 to March 2015) home visits, with a mean (SD) follow-up interval of 7.6 (1.4) years. By design, approximately half of the included women had been diagnosed and treated for breast cancer after enrollment and before the second blood draw. A total of 410 women were included in the present study, including 185 breast cancer survivors and 225 who remained free of breast cancer over a comparable follow-up period. Data were analyzed from April 21 to September 9, 2022.

**Exposures:**

Breast cancer status and, among breast cancer survivors, cancer treatment type (chemotherapy, radiotherapy, endocrine therapy, or surgery).

**Main Outcomes and Measures:**

Blood DNA methylation data were generated in 2019 using a genome-wide methylation screening tool and deconvolved to estimate percentages of 12 circulating leukocyte subsets.

**Results:**

Of the 410 women included in the analysis, the mean (SD) age at enrollment was 56 (9) years. Compared with breast cancer–free women, breast cancer survivors had decreased percentages of circulating eosinophils (−0.45% [95% CI, −0.87% to −0.03%]; *P* = .03), total CD4^+^ helper T cells (−1.50% [95% CI, −2.56% to −0.44%]; *P* = .01), and memory B cells (−0.22% [95% CI, −0.34% to −0.09%]; *P* = .001) and increased percentages of circulating naive B cells (0.46% [95% CI, 0.17%-0.75%]; *P* = .002). In breast cancer survivor–only analyses, radiotherapy was associated with decreases in total CD4^+^ T cell levels, whereas chemotherapy was associated with increases in naive B cell levels. Surgery and endocrine therapy were not meaningfully associated with leukocyte changes.

**Conclusions and Relevance:**

In this cohort study of 410 women, breast cancer survivors experienced lasting changes in peripheral leukocyte composition compared with women who remained free of breast cancer. These changes may be related to treatment with chemotherapy or radiotherapy and could influence future chronic disease risk.

## Key Points

**Question:**

Is a breast cancer diagnosis and treatment associated with long-term changes to peripheral immune cell composition, and if so, are these changes associated with different treatment modalities?

**Findings:**

In this cohort study of 410 participants, breast cancer survivors had lasting alterations to peripheral leukocyte composition compared with women who remained free of breast cancer; associations appeared to be driven by radiotherapy and chemotherapy.

**Meaning:**

These findings suggest that breast cancer survivors experience changes in peripheral leukocyte composition that are detectable years after diagnosis and may be associated with treatment.

## Teaser

This cohort study investigates whether breast cancer diagnosis and treatment are associated with lasting changes to the peripheral immune system.

## Introduction

Approximately 4 million women with a history of breast cancer (hereinafter referred to as breast cancer survivors) live in the US.^1^ Breast cancer survivors have higher age-specific rates of hypertension and other chronic diseases than women without a history of breast cancer.^2,3,4,5,6,7^ Our group^8^ and others^9,10,11^ have shown that alterations to the immune system are associated with risk of these chronic conditions in the general population, leading to the hypothesis that the higher chronic disease risk in breast cancer survivors may be driven, in part, by changes in immune function. Herein, we examine this hypothesis by investigating whether breast cancer diagnosis and treatment are associated with lasting changes to the peripheral immune system.

We have previously reported that circulating leukocyte composition becomes altered in the years leading up to a clinical diagnosis of breast cancer,^12^ and others^13,14,15^ have reported that leukocyte composition in blood samples differs between women diagnosed but not yet treated for breast cancer and those without breast cancer. Currently, little is known about the effects of different breast cancer treatments on leukocyte composition and the persistence of those possible effects over time. These questions can be best examined using prospective studies that compare longitudinal changes in leukocyte composition between breast cancer survivors and women who remain free of breast cancer. Because changes in leukocyte composition have been observed before diagnosis and treatment,^12,13,14,15^ investigations need to account for prediagnostic differences when characterizing treatment effects on circulating leukocyte composition.

Detailed leukocyte composition measurement has traditionally depended on flow cytometry of fresh blood samples.^16^ Compositional data are therefore rarely available in large epidemiologic cohorts, which typically bank frozen blood or DNA samples for future analyses. A recently developed technique called *methylation cytometry* solves this problem by using cell-type–specific patterns of DNA methylation (DNAm) to provide high-resolution estimates of leukocyte composition.^17,18,19,20,21^ Herein, we apply this technique to compare paired sets of blood samples collected a mean (SD) of 7.6 (1.4) years apart from women who did and did not develop breast cancer in the years between blood draws. Specifically, we aimed to investigate whether breast cancer and associated treatments are associated with lasting changes in leukocyte profiles.

## Methods

### Study Population

The Sister Study enrolled a nationwide, prospective cohort of 50 884 women living in the US (including Puerto Rico) between July 2003 and March 2009.^22^ The study was primarily designed to identify biological and environmental factors associated with breast cancer incidence and survival, and enrollment was therefore restricted to self-identified female participants. Women were eligible for enrollment if they were aged between 35 and 74 years, had a sister (full or half) diagnosed with breast cancer, and had not themselves been diagnosed with breast cancer. Baseline data were collected between 2003 and 2009 using self-completed questionnaires and a 2-part computer-assisted telephone interview. As part of enrollment, trained medical examiners conducted an in-person home visit to perform a biometric assessment and collect house dust and biospecimen samples, including whole blood. Participants were recontacted annually to collect new information on health (including incident breast cancer diagnoses) and every 2 to 3 years to update environmental exposure and lifestyle information; annual response rates have been approximately 90%. This study followed the Strengthening the Reporting of Observational Studies in Epidemiology (STROBE) reporting guideline. Written informed consent was collected at the home visit, and the Institutional Review Board of the National Institutes of Health approved and oversees the study. Additional information and procedures for accessing The Sister Study data are provided on the study website.^23^

Between October 2013 and March 2015, 3738 women who had provided a blood sample at the enrollment home visit were invited to participate in a second home visit.^24^ By design, half of the women were contacted because they had been diagnosed and treated for breast cancer after enrollment. Of the contacted women, 2315 (62%) provided a second whole blood sample, including 1146 breast cancer survivors and 1169 women who had remained free of breast cancer.

### DNAm Assessment and Quality Control

In 2019, paired blood samples collected at the baseline and follow-up home visits from 433 participants were selected for DNAm profiling. Approximately one-half of the women (197 [45%]) were selected from those diagnosed and treated for breast cancer between the blood draws, whereas the other half (236 [55%]) were selected from those who remained free of breast cancer. To investigate potential differences in association by race and ethnicity, women who self-reported as Black (Hispanic or non-Hispanic) were oversampled for both groups; members of racial and ethnicity minority groups comprised approximately 30% of the sample population.

Genomic DNA was extracted from whole blood aliquots as previously described.^25^ One microgram of extracted DNA was bisulfite converted using a commercially available kit (EZ DNA Methylation Kit; Zymo Research) in 96-well plates. Samples were tested for completeness of bisulfite conversion, and converted DNA was assayed using a genome-wide methylation screening tool (Infinium MethylationEPIC v1 BeadChip Array; Illumina) at the Cancer Genomics Research Laboratory of the National Cancer Institute.^26^

We used the ENmix pipeline^27^ for DNAm data preprocessing, as it is reported to outperform alternative pipelines.^28,29,30,31,32,33^ Samples were excluded if they did not meet quality control measures, including bisulfite intensity less than 5000, had greater than 5% of probes with low-quality methylation values (detection *P* > .000001, <3 beads, or values outside 3 × the IQR), or were outliers for their methylation beta value distributions. Of the 433 women in the sample, 12 were excluded because one of their samples failed DNAm quality control and another 11 were excluded because 1 or more of their samples were an extreme outlier in 1 or more of the leukocyte subset percentages (defined as outside 4 SDs from the population mean). In total, 410 women (185 cases with breast cancer and 225 breast cancer–free controls) remained in the analytic sample.

### Leukocyte Composition Estimation

Proportions of 12 circulating leukocyte subsets were estimated using the EPIC IDOL (identifying optimal DNA libraries) extended library for the MethylationEPIC v1 BeadChip.^20^ The leukocyte subsets in this library included those from both the myeloid (ie, neutrophil, basophil, eosinophil, and monocyte) and lymphoid (ie, naive and memory B cells, naive and memory CD4^+^ and CD8^+^ T cells, natural killer cells, and T regulatory cells) lineages. The leukocyte percentages were collapsed into higher-order subsets based on their hematopoietic lineage: neutrophils, basophils, and eosinophils were combined to represent total percentage of granulocytes; naive and memory subsets of B cells and CD8^+^ cytotoxic T cells were combined to represent total percentages of those subsets; and naive, memory, and T regulatory subsets were combined to represent total percentage of CD4^+^ helper T cells.

### Breast Cancer Characteristics and Therapies

Incident breast cancers and dates of diagnosis of The Sister Study participants were determined via follow-up questionnaire or other self-reports. Medical records were retrieved for 1112 women with breast cancer (97%) who provided a second blood draw, including nearly all women who were selected for DNAm profiling. Medical records abstraction retrieved information on breast cancer characteristics (estrogen receptor [ER] status and tumor invasiveness) and treatments (chemotherapy, radiotherapy, endocrine therapy, and surgery). In the analysis of treatments, survivors missing information on breast cancer therapies were excluded (n = 3).

### Statistical Analysis

Data were analyzed from April 21 to September 9, 2022. Characteristics of the sample population at baseline and follow-up, stratified by breast cancer status, were reported using means and SDs for continuous measures and counts and percentages for categorical measures.

Associations between breast cancer status and leukocyte subsets were examined using linear mixed-effects models. In all analyses, the leukocyte subset measures were treated as the dependent variable and breast cancer status was treated as the independent variable. All models were adjusted for chronological age (in years), and self-reported race and ethnicity (Hispanic or non-Hispanic Black and non-Hispanic White). A supplemental analysis was conducted using a fractional multinomial logistic regression model that treats the leukocyte subset percentages at the follow-up visit simultaneously as the dependent variables.^34^ This model was adjusted for the leukocyte percentage values at the baseline visit, self-reported race and ethnicity, baseline age, and follow-up time. Associations of breast cancer status with the leukocyte subset measures were also stratified by self-reported race and ethnicity, tumor invasiveness (ductal carcinoma in situ vs invasive carcinoma) and ER status (ER positive vs ER negative). Statistical tests for interaction were performed using 3-way interaction terms of breast cancer status, time, and the characteristic of interest (ie, self-reported race and ethnicity, tumor invasiveness, and ER status). Among breast cancer survivors, Pearson correlation coefficients were estimated for the changes in the leukocyte percentages between the blood draws and time since diagnosis.

In the analysis of breast cancer treatments, cancer-free participants were first compared with breast cancer survivors who were treated with surgery only. Subsequent analyses to determine associations with individual treatment classes were conducted using a survivor-only design. Specifically, linear mixed-effects models were constructed with leukocyte percentages measures as the dependent variable and with time interaction variables with chemotherapy (yes or no), radiotherapy (yes or no), or endocrine therapies (yes or no) included together as independent variables. In addition to the covariates listed above, treatment analyses were adjusted for tumor stage and ER status. Statistical significance was determined at 2-sided *P* ≤ .05, and all analyses were conducted using Stata, version 17 (StataCorp LLC).

## Results

In this longitudinal study of 410 women, the mean (SD) age at enrollment was 56 (9) years. A total of 123 women (30%) self-identified as Hispanic or non-Hispanic Black and 287 (70%) as non-Hispanic White. Compared with women who remained free of breast cancer, breast cancer survivors were slightly older at enrollment (Table). Overall, the mean (SD) time between blood draws was 7.6 (1.4) years. At baseline, 279 of 377 women (74%) in both groups with data available were postmenopausal and, by the time of the second blood draw, 360 (95%) were postmenopausal. Distributions of the leukocyte components at the baseline and follow-up blood draws by breast cancer status are displayed in eFigure 1 in Supplement 1. In the time between the blood draws, both breast cancer survivors and breast cancer–free women had statistically significant increases in the percentages of granulocytes (1.55% and 2.70%, respectively) and decreases in the percentages of naive CD8^+^ (−0.43% and −0.35%, respectively) and CD4^+^ (−0.70% and −1.77%, respectively) T cells and memory B cells (−0.09% and −0.30%, respectively) (eTable 1 in Supplement 1).

**Table.** Characteristics of the Sample Population at Baseline (2003-2009) and Follow-Up (2013-2015)

Characteristic	Breast cancer–free controls (n = 225)		Breast cancer survivors (n = 185)	
	Baseline	Follow-up	Baseline	Follow-up
Age, mean (SD), y	55 (9)	63 (9)	57 (9)	65 (9)
Body mass index, mean (SD)^a^	28 (7)	29 (7)	29 (6)	29 (6)
Physical activity, mean (SD), h/wk	13 (8)	10 (16)	13 (7)	9 (11)
Alcohol intake, mean (SD), drinks/wk	2.3 (4)	2.9 (5)	1.8 (3)	3.0 (5)
Smoking history, mean (SD), pack-years	4.8 (10)	4.9 (10)	6.6 (15)	6.8 (15)
Stress, mean (SD)^b^	2.7 (3)	3.9 (3)	2.3 (3)	3.4 (3)
Follow-up, mean (SD), y		7.5 (1)		7.8 (1)
Menopause status, No. (%)^c^^,^^d^				
Premenopausal	76 (34)	7 (4)	55 (30)	10 (5)
Postmenopausal	149 (66)	185 (96)	130 (70)	175 (95)
Self-reported race and ethnicity, No. (%)^d^				
Black (Hispanic or non-Hispanic)	78 (35)	NA	45 (24)	NA
Non-Hispanic White	147 (65)	NA	140 (76)	NA
Educational attainment, No. (%)^d^				
High school, GED, or less	31 (14)	NA	22 (12)	NA
Some college	69 (31)	NA	50 (27)	NA
College graduate or more	125 (56)	NA	113 (61)	NA

Abbreviations: GED, General Educational Development; NA, not applicable.

^a^ Calculated as weight in kilograms divided by height in meters squared.

^b^ Measured using the short version of the Perceived Stress Scale to assess the participant’s internal appraisal of stress in the last 30 days. Scores range from 0 to 16, with higher scores indicating higher levels of perceived stress.

^c^ Thirty-three breast cancer–free controls were missing menopause status at the follow-up visit.

^d^ Percentages have been rounded and may not total 100.

Among the breast cancer survivors, diagnoses occurred a mean (SD) of 3.6 (2.0) years after the initial blood draw and 4.2 (1.9) years before the second blood draw (eFigure 2 in Supplement 1). Most tumors were ER positive, and most were classified as invasive (eTable 2 in Supplement 1). Nearly all women (180 of 185 [97%]) were treated with surgery; in addition, 126 (68%) received endocrine therapy, 118 (64%) received radiotherapy, and 65 (35%) received chemotherapy (eTable 2 in Supplement 1).

### Breast Cancer Status and Changes in Leukocyte Composition in the Interval Between Blood Draws

**Figure 1.**
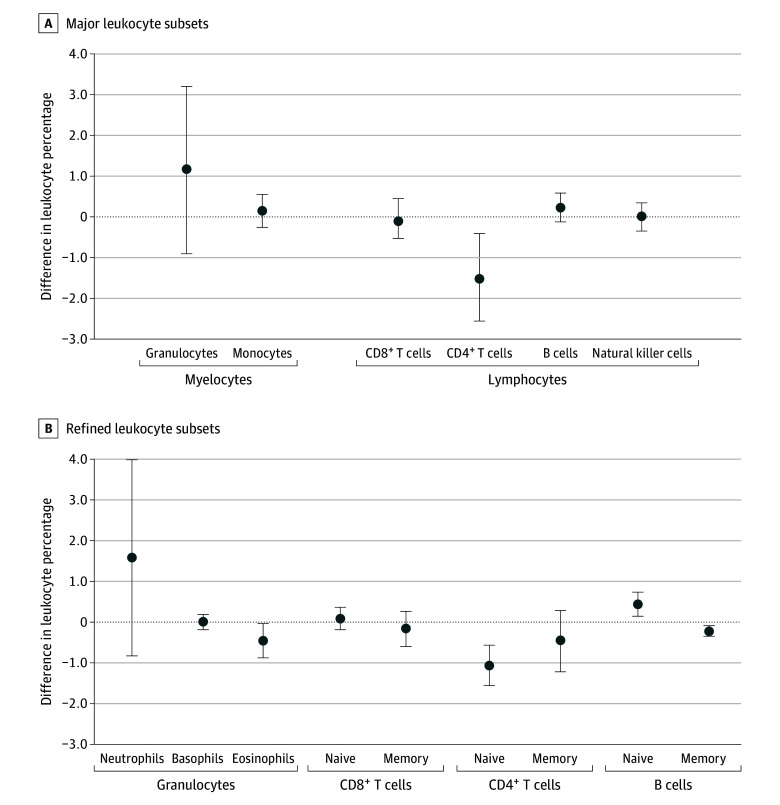
Analysis of Circulating Leukocyte Composition and Breast Cancer Status Results are from separate mixed-effects linear regression models treating breast cancer status as an independent variable and the 6 major leukocyte subsets or the 9 refined subsets as the dependent variables. The reference group consisted of the women who remained free of breast cancer. Models were adjusted for race and ethnicity and age as fixed effects. Error bars indicate 95% CIs.

Compared with women who remained free of breast cancer, women diagnosed with breast cancer had decreases in the circulating percentage of total CD4^+^ helper T cells (−1.50% [95% CI, −2.56% to −0.44%]; *P* = .01) (Figure 1A). The decreases in CD4^+^ T cell levels appeared to be driven by reductions in the naive component (−1.05% [95% CI, −1.54% to −0.55%]; *P* < .001) (Figure 1B). Breast cancer survivors also had decreases in eosinophil (−0.45% [95% CI, −0.87% to −0.03%]; *P* = .03) and memory B cell (−0.22% [95% CI, −0.34% to −0.09%]; *P* = .001) levels and increases in naive B cell levels (0.46% [95% CI, 0.17%-0.75%]; *P* = .002) (Figure 1B). Sensitivity analyses using an alternate model (eTable 3 in Supplement 1) and stratification by self-reported race (eTable 4 in Supplement 1), tumor invasiveness (eTable 5 in Supplement 1), and tumor ER status (eTable 6 in Supplement 1) produced largely similar results. Among survivors, changes in leukocyte percentages between the first and second blood draw were not associated with the number of years between diagnosis and second blood draw (eFigure 3 in Supplement 1).

### Breast Cancer Therapies and Changes in Leukocyte Composition

Compared with cancer-free women, treatment of breast cancer survivors with only surgery was not associated with differences in any of the leukocyte subsets (eTable 7 in Supplement 1). However, nearly all breast cancer survivors treated with only surgery (36 of 40 [90%]) were diagnosed with ductal carcinoma in situ.

**Figure 2.**
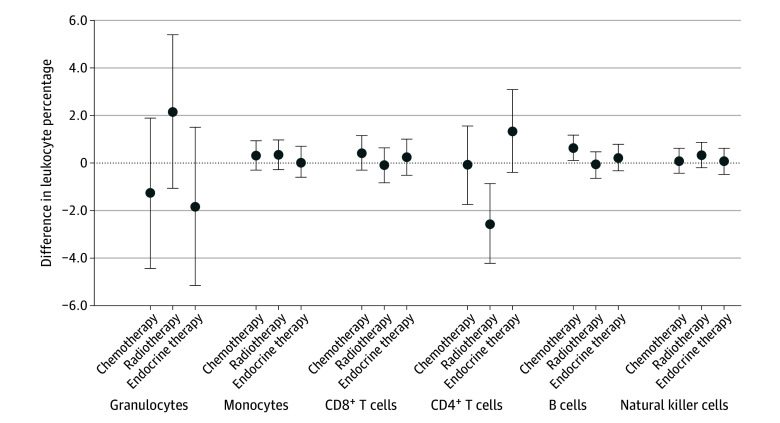
Breast Cancer Survivor–Only Analysis of the 6 Primary Leukocyte Subsets and Nonsurgical Breast Cancer Therapies Results are from separate mixed-effects linear regression simultaneously including the 3 treatment classes (chemotherapy, radiotherapy, and endocrine therapy) as independent variables and the 6 major leukocyte subsets as dependent variables. Models were adjusted for race and ethnicity, age, disease stage, and tumor receptor status as fixed effects. Error bars indicate 95% CIs.

**Figure 3.**
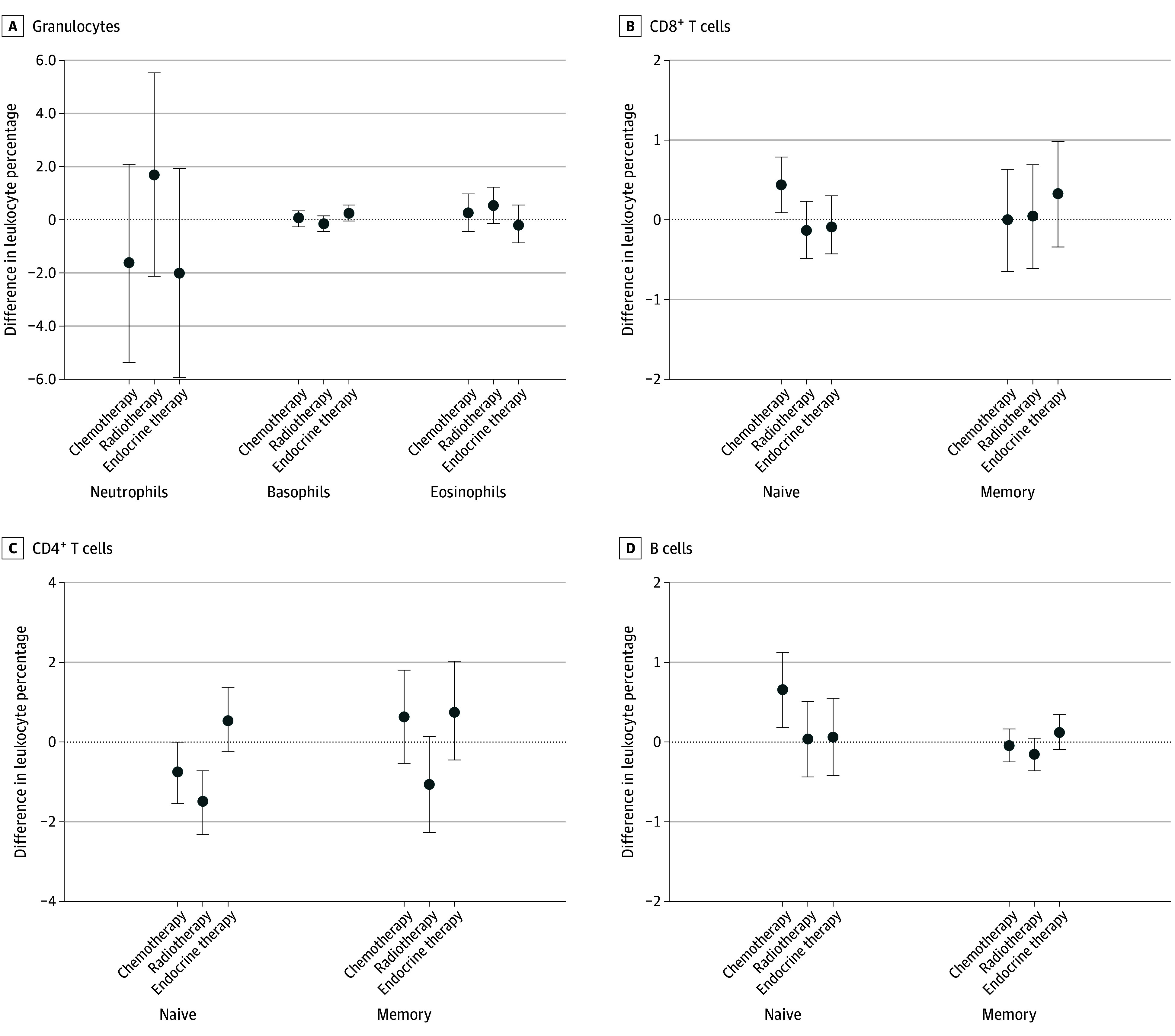
Survivor-Only Analysis of the 9 Leukocyte Component Subsets and Nonsurgical Breast Cancer Therapies Results are from separate mixed-effects linear regression simultaneously including the 3 treatment classes (chemotherapy, radiotherapy, and endocrine therapy) as independent variables and the granulocyte components, CD8^+^ T-cell components, CD4^+^ T-cell components, and B-cell components as dependent variables. Models were adjusted for race and ethnicity, age, disease stage, and tumor receptor status as fixed effects. Error bars indicate 95% CIs.

In a breast cancer survivor–only analysis using models that simultaneously included variables for each of the 3 types of nonsurgical therapies, endocrine therapy did not show statistically significant associations with changes in any of the leukocyte subtypes (Figure 2 and eTable 8 in Supplement 1). In contrast, radiotherapy was associated with decreases in circulating percentage of total CD4^+^ helper T cells, and chemotherapy was associated with increases in percentage of total B cells (Figure 2 and eTable 8 in Supplement 1). In an analysis of the naive and memory component subsets, radiotherapy was associated with decreased percentages of both the naive and memory components of CD4^+^ T cells (Figure 3 and eTable 8 in Supplement 1), whereas chemotherapy was associated with increased percentages of only the naive components of CD8^+^ T and B cells (Figure 3 and eTable 8 in Supplement 1).

## Discussion

In this cohort study, we used paired blood samples collected a mean of 7.6 years apart from women who did and did not develop breast cancer and examined whether an intervening breast cancer diagnosis and treatment was associated with changes in circulating leukocyte composition. Compared with women who remained free of breast cancer over the interval, breast cancer survivors had lasting decreases in the circulating percentages of various leukocyte subsets, including eosinophils, T cell, and B cells. In the analysis of different breast cancer therapies, radiotherapy was associated with decreases in percentages of total CD4^+^ T cells, and chemotherapy was associated with increases in percentages of naive B cells.

Breast cancer survivors are at higher risk than the general population for various chronic diseases.^2,3,4,5,6,7^ These risks may be associated with the types of breast cancer therapies received. Compared with the general population, women treated with radiotherapy experience higher rates of hypertension, cardiac arrest, and heart failure and/or cardiomyopathy; women treated with chemotherapy experience higher rates of type 2 diabetes, arrhythmia, and heart failure and/or cardiomyopathy.^3,4^ Changes in the peripheral immune system are associated with the incidence of many of these conditions,^8,9,10,11^ and cancer therapies have been reported to alter leukocyte composition in the weeks to months after treatment.^35,36,37,38,39,40,41^ Herein, we extend this work by finding that radiotherapy and chemotherapy may differentially alter specific leukocyte subsets and that these changes evidently persist for years after diagnosis.

Existing studies of leukocyte composition^42,43,44,45,46,47^ have largely focused on acute effects of therapy. A notable feature of our study is the length of follow-up whereby paired blood samples were collected nearly a decade apart from women who did and did not develop breast cancer in the interval between the collections. Estimation of leukocyte composition, which has traditionally been accomplished by flow cytometry, was made possible with the advent of methylation cytometry.^17,18,19,20,21^ Notably, the interval associated–related directional changes in leukocyte subsets observed in the breast cancer–free women in this study were similar to those previously reported from the Women’s Health Initiative,^48^ lending validity to the methylation cytometry technique.

In the years following diagnosis, breast cancer survivors experienced decreases in circulating percentages of CD4^+^ helper T cells. Prior studies in patients with breast cancer^41,42,43^ have reported acute reductions in CD4^+^ helper T cell levels in the weeks to months after chemotherapy. In a study of long-term effects, incomplete recovery of naive CD4^+^ helper T cells was reported 1 to 5 years after chemotherapy.^49^ We also observed lasting decreases in naive CD4^+^ helper T cell levels in breast cancer survivors and found these changes were most pronounced among women with a history of radiotherapy. In experimental studies, T cells undergo apoptosis at higher rates than other leukocytes after low-dose radiation exposure,^50^ with CD4^+^ helper T cells being more radiosensitive than CD8^+^ cytotoxic T cells.^51^ Naive T-cell components are also reported to replenish at slower rates than memory components,^52^ supporting the hypothesis that naive CD4^+^ T cells may be particularly susceptible to radiation therapy.

We also observed that women diagnosed with and treated for breast cancer showed lasting increases in circulating B-cell percentage, with increases in naive B-cell components outweighing a small decrease in memory components. Prior studies investigating the effects of breast cancer therapies on B-cell populations^41,42,43,53^ have had inconsistent findings. Decreases in B-cell levels have been reported in the weeks to months following chemotherapy,^41,42,43^ whereas increases have been reported in survivors up to 2 years after treatment.^53^ Few studies have information on the naive and memory components of B cells. In our study, chemotherapy was positively associated with B-cell percentages, which appears largely a result of increases in naive B-cell components. Although the overall hematological toxic effects of chemotherapy and low-dose radiation are reported to be similar,^54^ our observations suggest that these therapy types may differentially affect leukocyte subsets.

Treatment-associated immunological changes have important implications for long-term health among survivors of breast cancer. Breast cancer survivors are at an 80% higher risk of dying of cardiovascular disease than women in the general population, with the greatest relative risk occurring nearly a decade after diagnosis.^55^ Hypertension is an important precursor to cardiovascular disease, and survivors of breast cancer treated with radiation are at increased risk.^3^ Our group^8^ has previously shown that lower circulating percentages of naive CD4^+^ helper T cells and higher percentages of B cells are associated with a 20% higher risk of developing hypertension. In the present study, we observed these same shifts in breast cancer survivors relative to cancer-free participants, with lower CD4^+^ helper T-cell levels seen after radiation exposure and higher B-cell levels seen after chemotherapy. These observations lend support to the hypothesis that the increased risk of cardiovascular disease in breast cancer survivors is due, in part, to lasting changes in immunity.

### Limitations

This study has some limitations. All women enrolled in The Sister Study cohort have a first-degree family history of breast cancer. In addition, the study sample size limited our ability to examine the details of treatment-related factors. Methylation cytometry estimates the percentages of different leukocyte subsets that can be converted to cell counts if total white blood cell counts are assayed at the time of blood draw, but cell count data were not available in our study. Although we found little evidence of difference between cancer-free participants and breast cancer survivors treated with surgery only or with endocrine therapy, we cannot rule out the possibility that the observed changes in leukocyte composition are driven by postdiagnosis changes in non–treatment-related factors such as stress and lifestyle.^24,56^

## Conclusions

In this cohort study, we found that survivors of breast cancer had lasting changes in peripheral leukocyte composition that may be associated with the types of treatments received. These findings add to our understanding of the biological changes that underlie the long-term health of breast cancer survivors.

## Notes

Supplement 1. **eFigure 1.** Distributions of the Circulating Leukocyte Subsets at Baseline and Follow-Up by Breast Cancer Status **eTable 1.** Changes in the Leukocyte Subsets Between the Baseline and Follow-Up Blood Draws, Stratified by Breast Cancer Status **eFigure 2.** Time to Breast Cancer Diagnosis From Baseline Blood Draw **eTable 2.** Tumor Subtype and Therapies Received Among Women Diagnosed With Breast Cancer, Overall and Stratified by Self-Reported Race and Ethnicity **eTable 3.** Comparison of Statistical Techniques for Analysis of Changes in Circulating Leukocyte Composition and Breast Cancer Status **eTable 4.** Linear Mixed-Effect Associations Between All Leukocyte Subsets and Breast Cancer Status Stratified by Self-Reported Race **eTable 5.** Linear Mixed-Effect Associations Between All Leukocyte Subsets and Breast Cancer Status, Stratified by Tumor Invasiveness **eTable 6.** Linear Mixed-Effect Associations Between All Leukocyte Subsets and Breast Cancer Status, Stratified by Tumor Estrogen Receptor Status **eFigure 3.** Pearson Correlation Between Changes in the Leukocyte Subsets Between the Blood Draws and Time Since Diagnosis **eTable 7.** Linear Mixed-Effect Associations Between All Leukocyte Subsets and Breast Cancer Status, Restricted to Survivors Treated With Surgery Only **eTable 8.** Linear Mixed-Effect Associations Between All Leukocyte Subsets and Breast Cancer Treatments

Supplement 2.
Data Sharing Statement
