# Supplementary material for: Circulating Leukocyte Subsets Before and After a Breast Cancer Diagnosis and Therapy
Source: JAMA Netw Open. 2024 Feb 15;7(2):e2356113. doi: 10.1001/jamanetworkopen.2023.56113 (PMC10870180; doi:10.1001/jamanetworkopen.2023.56113)
Supplement: Supplement 1. — eFigure 1. Distributions of the Circulating Leukocyte Subsets at Baseline and Follow-Up by Breast Cancer Status eTable 1. Changes in the Leukocyte Subsets Between the Baseline and Follow-Up Blood Draws, Stratified by Breast Cancer Status eFigure 2. Time to Breast Cancer Diagnosis From Baseline Blood Draw eTable 2. Tumor Subtype and Therapies Received Among Women Diagnosed With Breast Cancer, Overall and Stratified by Self-Reported Race and Ethnicity eTable 3. Comparison of Statistical Techniques for Analysis of Changes in Circulating Leukocyte Composition and Breast Cancer Status eTable 4. Linear Mixed-Effect Associations Between All Leukocyte Subsets and Breast Cancer Status Stratified by Self-Reported Race eTable 5. Linear Mixed-Effect Associations Between All Leukocyte Subsets and Breast Cancer Status, Stratified by Tumor Invasiveness eTable 6. Linear Mixed-Effect Associations Between All Leukocyte Subsets and Breast Cancer Status, Stratified by Tumor Estrogen Receptor Status eFigure 3. Pearson Correlation Between Changes in the Leukocyte Subsets Between the Blood Draws and Time Since Diagnosis eTable 7. Linear Mixed-Effect Associations Between All Leukocyte Subsets and Breast Cancer Status, Restricted to Survivors Treated With Surgery Only eTable 8. Linear Mixed-Effect Associations Between All Leukocyte Subsets and Breast Cancer Treatments [file jamanetwopen-e2356113-s001.pdf]

# Supplementary Online Content

Kresovich JK, O'Brien KM, Xu Z, Weinberg CR, Sandler DP, Taylor JA. Circulating leukocyte subsets before and after a breast cancer diagnosis and therapy. *JAMA Netw Open*. 2024;7(2):e2356113. doi:10.1001/jamanetworkopen.2023.56113

**eFigure 1.** Distributions of the Circulating Leukocyte Subsets at Baseline and Follow-Up by Breast Cancer Status

**eTable 1.** Changes in the Leukocyte Subsets Between the Baseline and Follow-Up Blood Draws, Stratified by Breast Cancer Status

**eFigure 2.** Time to Breast Cancer Diagnosis From Baseline Blood Draw

**eTable 2.** Tumor Subtype and Therapies Received Among Women Diagnosed With Breast Cancer, Overall and Stratified by Self-Reported Race and Ethnicity

**eTable 3.** Comparison of Statistical Techniques for Analysis of Changes in Circulating Leukocyte Composition and Breast Cancer Status

**eTable 4.** Linear Mixed-Effect Associations Between All Leukocyte Subsets and Breast Cancer Status Stratified by Self-Reported Race

**eTable 5.** Linear Mixed-Effect Associations Between All Leukocyte Subsets and Breast Cancer Status, Stratified by Tumor Invasiveness

**eTable 6.** Linear Mixed-Effect Associations Between All Leukocyte Subsets and Breast Cancer Status, Stratified by Tumor Estrogen Receptor Status

**eFigure 3.** Pearson Correlation Between Changes in the Leukocyte Subsets Between the Blood Draws and Time Since Diagnosis

**eTable 7.** Linear Mixed-Effect Associations Between All Leukocyte Subsets and Breast Cancer Status, Restricted to Survivors Treated With Surgery Only

**eTable 8.** Linear Mixed-Effect Associations Between All Leukocyte Subsets and Breast Cancer Treatments

This supplementary material has been provided by the authors to give readers additional information about their work.

© 2024 Kresovich JK et al. *JAMA Network Open*.

**eFigure 1.** Distributions of the Circulating Leukocyte Subsets at Baseline and Follow-Up by Breast Cancer Status

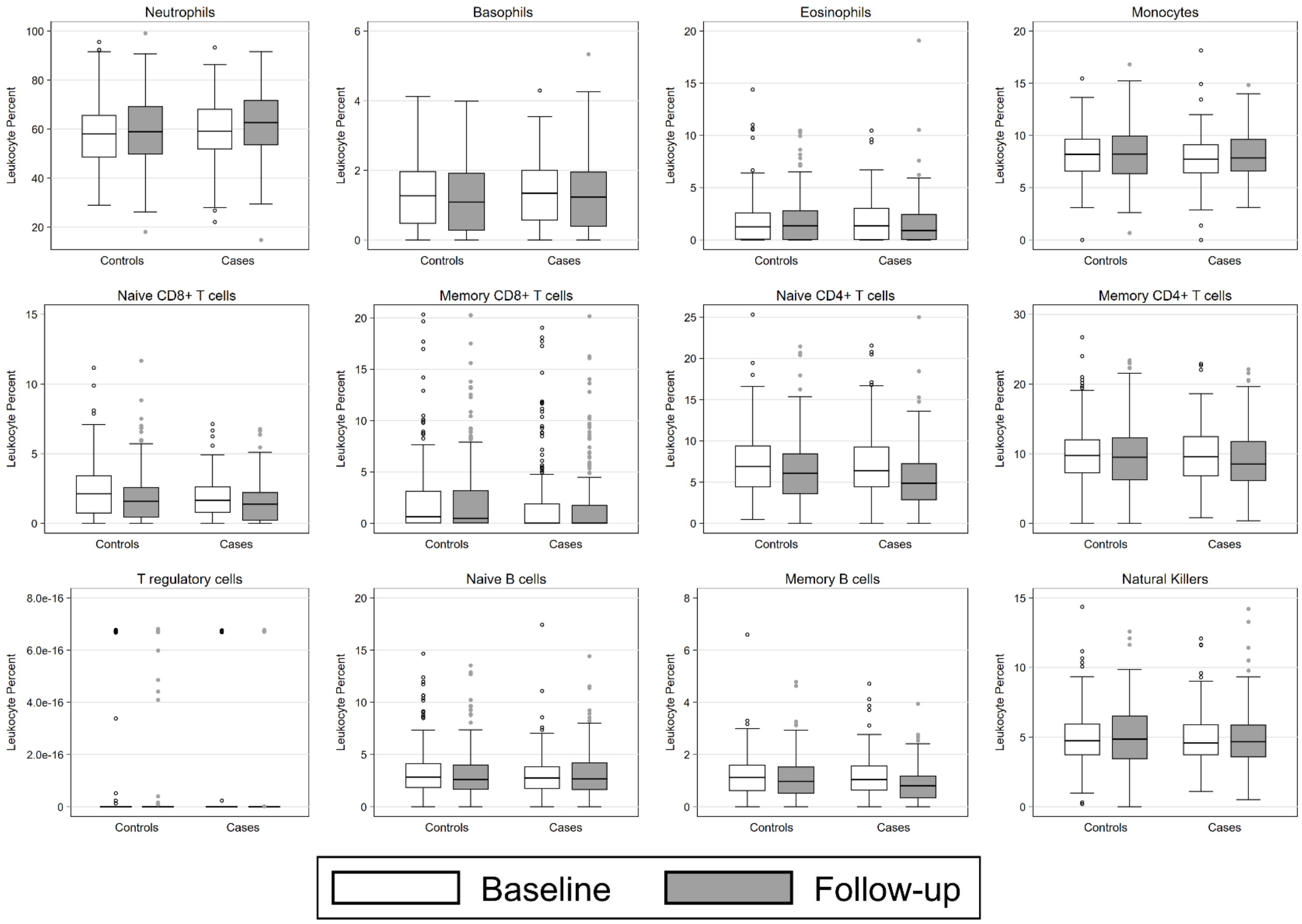


Box and whisker plots depicting the distributions of the twelve leukocyte subsets at baseline and follow-up separately for breast cancer survivors and women who remained breast cancer-free.

© 2024 Kresovich JK et al. *JAMA Network Open*.

**eTable 1.** Changes in the Leukocyte Subsets Between the Baseline and Follow-Up Blood Draws, Stratified by Breast Cancer Status

| | Breast cancer-free women | | Breast cancer survivors | |
|---|---|---|---|---|
| Leukocyte subset | Mean Difference | P-value | Mean Difference | P-value |
| Granulocytes | 1.55 | 0.03 | 2.7 | 0.001 |
| Neutrophils | 1.47 | 0.08 | 3.05 | 0.001 |
| Basophils | -0.09 | 0.12 | -0.08 | 0.24 |
| Eosinophils | 0.17 | 0.23 | -0.28 | 0.09 |
| Monocytes | 0.10 | 0.45 | 0.27 | 0.07 |
| CD8+ T cells | -0.33 | 0.06 | -0.39 | 0.03 |
| Naïve | -0.43 | < 0.001 | -0.35 | < 0.001 |
| Memory | 0.10 | 0.50 | -0.04 | 0.82 |
| CD4+ T cells | -0.97 | 0.01 | -2.48 | < 0.001 |
| Naïve | -0.70 | < 0.001 | -1.77 | < 0.001 |
| Memory | -0.27 | 0.27 | -0.72 | 0.01 |
| B cells | -0.36 | 0.002 | -0.12 | 0.37 |
| Naïve | -0.27 | 0.01 | 0.19 | 0.10 |
| Memory | -0.09 | 0.04 | -0.30 | < 0.001 |
| Natural Killers | -0.00 | 0.99 | 0.02 | 0.90 |

P-values calculated using paired T-test.

© 2024 Kresovich JK et al. *JAMA Network Open*.

**eFigure 2.** Time to Breast Cancer Diagnosis From Baseline Blood Draw

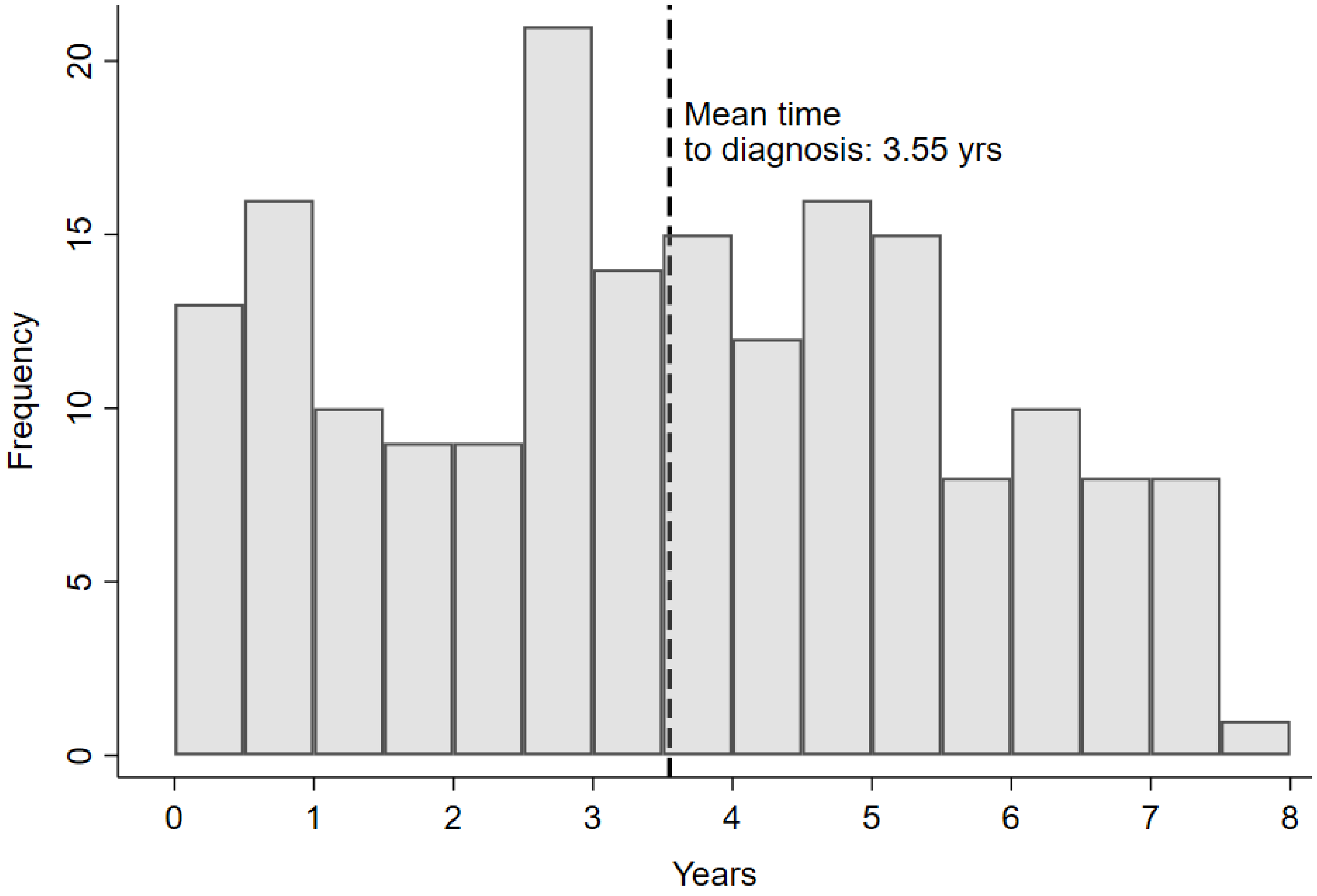


Histogram depicting the distribution for the time to diagnosis from the baseline blood draw among breast cancer survivor group (N= 185).

© 2024 Kresovich JK et al. *JAMA Network Open*.

**eTable 2.** Tumor Subtype and Therapies Received Among Women Diagnosed With Breast Cancer, Overall and Stratified by Self-Reported Race and Ethnicity

| Characteristic | Total N= 185 | Non-Hispanic White[1] N= 140 | Hispanic or non-Hispanic Black[2] N= 45 |
|---|---|---|---|
| Estrogen receptor status, N (%) | | | |
| Positive | 153 (83) | 119 (85) | 34 (76) |
| Negative | 32 (17) | 21 (15) | 11 (24) |
| Stage at diagnosis, N (%) | | | |
| 0 | 54 (29) | 41 (29) | 13 (29) |
| I | 89 (48) | 66 (47) | 23 (51) |
| II | 37 (20) | 29 (21) | 8 (18) |
| III | 5 (3) | 4 (3) | 1 (2) |
| Therapy received, N (%)[1] | | | |
| Surgery | 180 (97) | 137 (98) | 43 (96) |
| Any chemotherapy | 65 (35) | 45 (32) | 20 (44) |
| Any radiation | 118 (64) | 87 (62) | 31 (69) |
| Any endocrine therapy | 126 (68) | 102 (73) | 24 (53) |

[1]1 Black women missing information on surgery status; 3 women (2 White, 1 Black) missing information on receipt of endocrine, radiation or chemotherapy

© 2024 Kresovich JK et al. *JAMA Network Open*.

**eTable 3.** Comparison of Statistical Techniques for Analysis of Changes in Circulating Leukocyte Composition and Breast Cancer Status

| | Mixed effects linear regression models[1] | | | Fractional multinomial logistic regression model[2] | | |
|---|---|---|---|---|---|---|
| Leukocyte subsets | β | 95% CI | P-value | β | 95% CI | P-value |
| Granulocytes | 1.15 | -0.90, 3.20 | 0.27 | 0.88 | -0.94, 2.69 | 0.34 |
| Neutrophils | 1.59 | -0.81, 3.99 | 0.19 | 1.05 | -1.07, 3.19 | 0.42 |
| Basophils | 0.01 | -0.16, 0.18 | 0.93 | 0.10 | -0.06, 0.26 | 0.06 |
| Eosinophils | -0.45 | -0.87, -0.03 | 0.03 | -0.21 | -0.61, 0.18 | 0.20 |
| Monocytes | 0.16 | -0.23, 0.55 | 0.42 | 0.16 | -0.22, 0.53 | 0.42 |
| CD8+ T cells | -0.06 | -0.56, 0.43 | 0.80 | -0.53 | -1.26, 0.18 | 0.15 |
| Naïve | 0.09 | -0.16, 0.35 | 0.48 | 0.06 | -0.18, 0.30 | 0.83 |
| Memory | -0.16 | -0.58, 0.27 | 0.47 | -0.47 | -0.95, 0.02 | 0.06 |
| CD4+ T cells | -1.50 | -2.56, -0.44 | 0.01 | -1.17 | -2.11, -0.23 | 0.02 |
| Naïve | -1.05 | -1.54, -0.55 | < 0.001 | -1.18 | -1.64, -0.71 | < 0.001 |
| Memory | -0.45 | -1.18, 0.27 | 0.22 | 0.03 | -0.63, 0.68 | 0.78 |
| B cells | 0.24 | -0.10, 0.59 | 0.17 | 0.33 | 0.00, 0.66 | 0.05 |
| Naïve | 0.46 | 0.17, 0.75 | 0.002 | 0.57 | 0.24, 0.89 | 0.01 |
| Memory | -0.22 | -0.34, -0.09 | 0.001 | -0.19 | -0.31, -0.07 | 0.005 |
| Natural Killers | 0.01 | -0.32, 0.34 | 0.96 | 0.07 | -0.25, 0.39 | 0.65 |

[1] Results from separate mixed effect linear regression models treating breast cancer status as an independent variable and the six major leukocyte subsets as the dependent variables. Models adjusted for age and self-reported race.

[2] Results from a single fractional multinomial logistic regression model which account for the compositional nature of the leukocyte subsets as the dependent variables. The model treats breast cancer status as the independent variable and the leukocyte subset variables at the second time-point simultaneously as the dependent variables. Model adjusted for baseline age, follow-up time, self-reported race, and the baseline estimates of leukocyte composition. Models did not converge for the fractional multinomial logistic regression models for the refined leukocyte subsets. Analyses excluded T regulatory cells as nearly all predicted values were zero and inclusion prevented model conversion

© 2024 Kresovich JK et al. *JAMA Network Open*.

**eTable 4.** Linear Mixed-Effect Associations Between All Leukocyte Subsets and Breast Cancer Status Stratified by Self-Reported Race

| | White Women | | | Black Women | | | |
|---|---|---|---|---|---|---|---|
| Leukocyte subsets | β | 95% CI | P-value | β | 95% CI | P-value | P-int |
| Granulocytes | 1.62 | -0.85, 4.09 | 0.20 | -0.71 | -4.38, 2.95 | 0.70 | 0.32 |
| Neutrophils | 2.04 | -0.87, 4.96 | 0.17 | -0.21 | -4.45, 4.03 | 0.92 | 0.41 |
| Basophils | 0.03 | -0.17, 0.24 | 0.76 | -0.05 | -0.36, 0.25 | 0.74 | 0.66 |
| Eosinophils | -0.46 | -0.97, 0.06 | 0.08 | -0.45 | -1.18, 0.28 | 0.22 | 0.99 |
| Monocytes | 0.06 | -0.43, 0.54 | 0.82 | 0.59 | -0.07, 1.25 | 0.08 | 0.23 |
| CD8+ T cells | -0.20 | -0.74, 0.33 | 0.46 | 0.39 | -0.70, 1.48 | 0.49 | 0.30 |
| Naïve | 0.07 | -0.22, 0.35 | 0.65 | 0.13 | -0.41, 0.68 | 0.63 | 0.82 |
| Memory | -0.27 | -0.72, 0.19 | 0.25 | 0.25 | -0.69, 1.20 | 0.60 | 0.28 |
| CD4+ T cells | -1.54 | -2.83, -0.26 | 0.02 | -1.09 | -2.97, 0.79 | 0.26 | 0.71 |
| Naïve | -1.02 | -1.60, -0.44 | 0.001 | -1.15 | -2.09, -0.20 | 0.02 | 0.82 |
| Memory | -0.52 | -1.40, 0.36 | 0.24 | 0.06 | -1.22, 1.34 | 0.93 | 0.48 |
| B cells | 0.15 | -0.23, 0.52 | 0.45 | 0.49 | -0.27, 1.24 | 0.21 | 0.38 |
| Naïve | 0.33 | 0.04, 0.63 | 0.03 | 0.77 | 0.09, 1.44 | 0.03 | 0.19 |
| Memory | -0.19 | -0.34, -0.04 | 0.02 | -0.28 | -0.50, -0.05 | 0.02 | 0.52 |
| Natural Killers | -0.08 | -0.48, 0.32 | 0.71 | 0.34 | -0.24, 0.92 | 0.26 | 0.27 |

Results from separate mixed effect linear regression models treating breast cancer status as an independent variable and the six major leukocyte subsets as the dependent variables. Models adjusted for age and are stratified by self-reported race.
P-interaction, P-int.

© 2024 Kresovich JK et al. *JAMA Network Open*.

**eTable 5.** Linear Mixed-Effect Associations Between All Leukocyte Subsets and Breast Cancer Status, Stratified by Tumor Invasiveness

| | DCIS | | | Invasive | | | |
|---|---|---|---|---|---|---|---|
| Leukocyte subsets | β | 95% CI | P-value | β | 95% CI | P-value | P-int |
| Granulocytes | 0.26 | -2.81, 3.33 | 0.87 | 1.51 | -0.80, 3.81 | 0.20 | 0.67 |
| Neutrophils | 0.89 | -2.70, 4.48 | 0.63 | 1.87 | -0.83, 4.58 | 0.17 | 0.96 |
| Basophils | -0.06 | -0.32, 0.19 | 0.62 | 0.04 | -0.15, 0.23 | 0.70 | 0.63 |
| Eosinophils | -0.56 | -1.16, 0.03 | 0.06 | -0.40 | -0.88, 0.07 | 0.10 | 0.05 |
| Monocytes | -0.05 | -0.63, 0.54 | 0.88 | 0.24 | -0.19, 0.68 | 0.27 | 0.82 |
| CD8+ T cells | 0.20 | -0.58, 0.98 | 0.62 | -0.17 | -0.71, 0.38 | 0.55 | 0.99 |
| Naïve | -0.01 | -0.41, 0.40 | 0.98 | 0.13 | -0.16, 0.42 | 0.37 | 0.16 |
| Memory | 0.20 | -0.47, 0.87 | 0.56 | -0.30 | -0.76, 0.16 | 0.20 | 0.65 |
| CD4+ T cells | -0.64 | -2.23, 0.94 | 0.43 | -1.84 | -3.02, -0.66 | 0.002 | 0.24 |
| Naïve | -0.65 | -1.38, 0.08 | 0.08 | -1.20 | -1.74, -0.66 | < 0.001 | 0.21 |
| Memory | 0.00 | -1.06, 1.06 | 0.99 | -0.64 | -1.45, 0.18 | 0.13 | 0.79 |
| B cells | 0.16 | -0.33, 0.66 | 0.52 | 0.27 | -0.12, 0.66 | 0.17 | 0.29 |
| Naïve | 0.25 | -0.16, 0.66 | 0.24 | 0.54 | 0.22, 0.87 | 0.001 | 0.17 |
| Memory | -0.08 | -0.27, 0.10 | 0.38 | -0.27 | -0.41, -0.13 | < 0.001 | 0.35 |
| Natural Killers | 0.07 | -0.45, 0.58 | 0.80 | -0.02 | -0.37, 0.34 | 0.93 | 0.82 |

Results from separate mixed effect linear regression models treating breast cancer status as an independent variable and the six major leukocyte subsets as the dependent variables. Models adjusted for age and are stratified by self-reported race.
P-interaction, P-int.

© 2024 Kresovich JK et al. *JAMA Network Open*.

**eTable 6.** Linear Mixed-Effect Associations Between All Leukocyte Subsets and Breast Cancer Status, Stratified by Tumor Estrogen Receptor Status

| | ER negative | | | ER positive | | | |
|---|---|---|---|---|---|---|---|
| Leukocyte subsets | β | 95% CI | P-value | β | 95% CI | P-value | P-int |
| Granulocytes | 2.71 | -1.17, 6.60 | 0.17 | 0.83 | -1.34, 2.99 | 0.45 | 0.25 |
| Neutrophils | 2.99 | -1.57, 7.56 | 0.20 | 1.30 | -1.23, 3.84 | 0.31 | 0.48 |
| Basophils | 0.03 | -0.28, 0.35 | 0.83 | 0.00 | -0.18, 0.18 | 0.98 | 0.96 |
| Eosinophils | -0.31 | -1.16, 0.53 | 0.47 | -0.48 | -0.88, -0.07 | 0.02 | 0.13 |
| Monocytes | -0.10 | -0.84, 0.63 | 0.78 | 0.22 | -0.20, 0.63 | 0.31 | 0.60 |
| CD8+ T cells | -0.21 | -1.20, 0.78 | 0.68 | -0.03 | -0.55, 0.48 | 0.90 | 0.41 |
| Naïve | 0.12 | -0.39, 0.64 | 0.64 | 0.09 | -0.19, 0.36 | 0.53 | 0.36 |
| Memory | -0.33 | -1.19, 0.53 | 0.45 | -0.12 | -0.55, 0.31 | 0.58 | 0.60 |
| CD4+ T cells | -2.75 | -4.73, -0.77 | 0.006 | -1.24 | -2.36, -0.12 | 0.03 | 0.16 |
| Naïve | -1.91 | -2.84, -0.97 | < 0.001 | -0.87 | -1.37, -0.36 | 0.001 | 0.03 |
| Memory | -0.85 | -2.19, 0.50 | 0.22 | -0.37 | -1.14, 0.39 | 0.34 | 0.99 |
| B cells | 0.22 | -0.44, 0.88 | 0.51 | 0.25 | -1.11, 0.60 | 0.18 | 0.69 |
| Naïve | 0.49 | -0.07, 1.04 | 0.09 | 0.45 | 0.16, 0.75 | 0.003 | 0.48 |
| Memory | -0.27 | -0.50, -0.03 | 0.03 | -0.21 | -0.34, -0.07 | 0.002 | 0.34 |
| Natural Killers | 0.13 | -0.49, 0.76 | 0.67 | -0.02 | -0.37, 0.33 | 0.92 | 0.72 |

Results from separate mixed effect linear regression models treating breast cancer status as an independent variable and the six major leukocyte subsets as the dependent variables. Models adjusted for age and are stratified by self-reported race.

P-interaction, P-int.

© 2024 Kresovich JK et al. *JAMA Network Open*.

**eFigure 3.** Pearson Correlation Between Changes in the Leukocyte Subsets Between the Blood Draws and Time Since Diagnosis

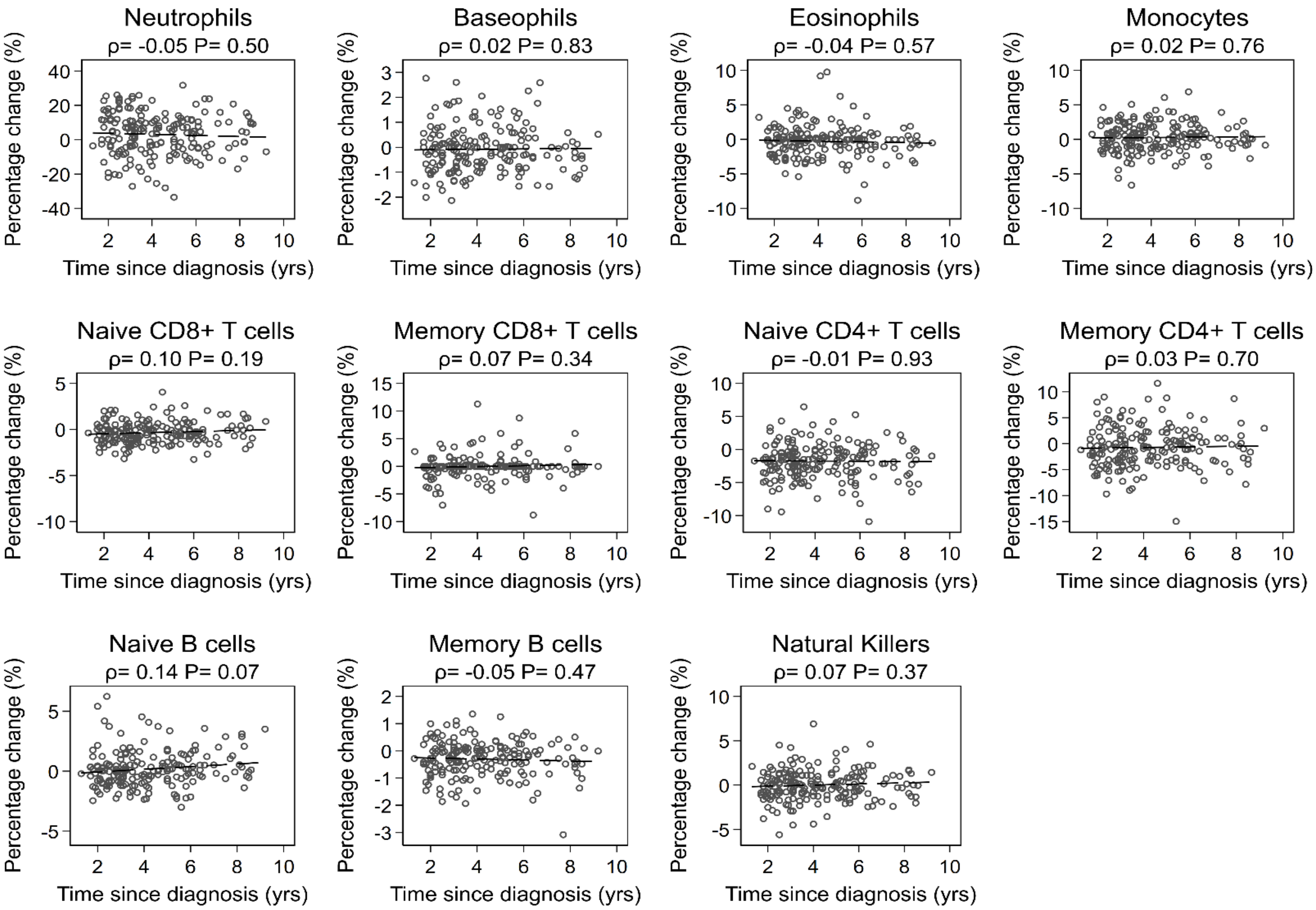


Among the breast cancer survivors, the Pearson correlation coefficient was calculated to examine the relationship between the changes the leukocyte subsets (excluding T-regulatory cells) between the blood draws and time since diagnosis.

© 2024 Kresovich JK et al. *JAMA Network Open*.

**eTable 7.** Linear Mixed-Effect Associations Between All Leukocyte Subsets and Breast Cancer Status, Restricted to Survivors Treated With Surgery Only

| Leukocyte subsets | β | 95% CI | P-value |
|---|---|---|---|
| Granulocytes | -0.51 | -5.30, 4.28 | 0.84 |
| Neutrophils | 0.21 | -5.40, 5.83 | 0.94 |
| Basophils | -0.30 | -0.69, 0.08 | 0.13 |
| Eosinophils | -0.42 | -1.34, 0.50 | 0.37 |
| Monocytes | -0.07 | -0.97, 0.82 | 0.87 |
| CD8+ T cells | -0.29 | -1.47, 0.88 | 0.62 |
| Naïve | 0.11 | -0.53, 0.75 | 0.74 |
| Memory | -0.40 | -1.40, 0.59 | 0.43 |
| CD4+ T cells | 0.75 | -1.71, 3.21 | 0.55 |
| Naïve | 0.36 | -0.74, 1.46 | 0.53 |
| Memory | 0.37 | -1.26, 2.05 | 0.64 |
| B cells | 0.18 | -0.62, 0.98 | 0.67 |
| Naïve | 0.39 | -0.27, 1.05 | 0.25 |
| Memory | -0.21 | -0.51, 0.09 | 0.16 |
| Natural Killers | -0.05 | -0.80, 0.71 | 0.90 |

Results from separate mixed effect linear regression models treating breast cancer status as an independent variable and the leukocyte subsets as the dependent variables. The breast cancer survivor sample was restricted to those treated with surgery only. Models adjusted for age and are stratified by self-reported race. Cancer-free women served as the referent group.

© 2024 Kresovich JK et al. *JAMA Network Open*.

**eTable 8.** Linear Mixed-Effect Associations Between All Leukocyte Subsets and Breast Cancer Treatments

| | Chemotherapy | | | Radiation | | | Endocrine therapy | | |
|---|---|---|---|---|---|---|---|---|---|
| Leukocyte subsets | β | 95% CI | P-value | β | 95% CI | P-value | β | 95% CI | P-value |
| Granulocytes | -1.30 | -4.47, 1.87 | 0.42 | 2.15 | -1.09, 5.39 | 0.19 | -1.86 | -5.19, 1.47 | 0.27 |
| Neutrophils | -1.62 | -5.36, 2.11 | 0.40 | -0.10 | -6.68, 6.47 | 0.98 | -1.98 | -5.90, 1.94 | 0.32 |
| Basophils | 0.06 | -0.21, 0.34 | 0.65 | -0.13 | -0.41, 0.15 | 0.37 | 0.26 | -0.03, 0.56 | 0.08 |
| Eosinophils | 0.26 | -0.42, 0.94 | 0.45 | 0.57 | -0.13, 1.26 | 0.11 | -0.14 | -0.86, 0.57 | 0.69 |
| Monocytes | 0.30 | -0.31, 0.92 | 0.33 | 0.32 | -0.31, 0.95 | 0.32 | 0.03 | -0.61, 0.68 | 0.92 |
| CD8+ T cells | 0.41 | -0.32, 1.13 | 0.27 | -0.11 | -0.85, 0.63 | 0.77 | 0.23 | -2.20, 0.99 | 0.56 |
| Naïve | 0.43 | 0.08, 0.78 | 0.02 | -0.14 | -0.50, 0.22 | 0.45 | -0.08 | -0.45, 0.29 | 0.66 |
| Memory | -0.02 | -0.66, 0.61 | 0.94 | 0.03 | -0.62, 0.68 | 0.94 | 0.31 | -0.35, 0.98 | 0.36 |
| CD4+ T cells | -0.11 | -1.77, 1.55 | 0.90 | -2.57 | -4.26, -0.88 | 0.003 | 1.34 | -0.40, 3.08 | 0.13 |
| Naïve | -0.76 | -1.52, 0.01 | 0.05 | -1.51 | -2.30, -0.73 | < 0.001 | 0.55 | -0.26, 1.35 | 0.18 |
| Memory | 0.65 | -0.52, 1.82 | 0.28 | -1.06 | -2.25, 0.14 | 0.08 | 0.79 | -0.43, 2.02 | 0.21 |
| B cells | 0.62 | 0.08, 1.16 | 0.03 | -0.12 | -0.67, 0.43 | 0.67 | 0.20 | -0.37, 0.77 | 0.49 |
| Naïve | 0.66 | 0.20, 1.12 | 0.005 | 0.04 | -0.43, 0.51 | 0.87 | 0.07 | -0.41, 0.55 | 0.78 |
| Memory | -0.04 | -0.24, 0.15 | 0.66 | -0.16 | -0.36, 0.04 | 0.12 | 0.13 | -0.08, 0.34 | 0.22 |
| Natural Killers | 0.08 | -0.45, 0.60 | 0.78 | 0.33 | -0.21, 0.87 | 0.23 | 0.06 | -0.40, 0.61 | 0.84 |

Results from separate mixed effect linear regression models treating breast cancer status as an independent variable and the six major leukocyte subsets as the dependent variables. Models adjusted for age and are stratified by self-reported race.

© 2024 Kresovich JK et al. *JAMA Network Open*.
