# Supplementary material for: Circulating Leukocyte Subsets Before and After a Breast Cancer Diagnosis and Therapy
Source: JAMA Netw Open. 2024 Feb 15;7(2):e2356113. doi: 10.1001/jamanetworkopen.2023.56113 (PMC10870180; doi:10.1001/jamanetworkopen.2023.56113)
Supplement: Supplement 2. — Data Sharing Statement [file jamanetwopen-e2356113-s002.pdf]

Data Sharing Statement

Kresovich. Circulating Leukocyte Subsets Before and After a Breast Cancer Diagnosis and Therapy. *JAMA Netw Open*. Published February 15, 2024.
doi:10.1001/jamanetworkopen.2023.56113

Data
Data available: Yes
Data types: Deidentified participant data
How to access data: Code and a limited dataset for replication purposes can be requested via the Sister Study website: https://sisterstudy.niehs.nih.gov/English/coll-data.htm.
When available: With publication

Supporting Documents
Document types: None

Additional Information
Who can access the data: Anyone interested
Types of analyses: Replication
Mechanisms of data availability: With approval from the Sister Study steering committee
